# Associations of cardiorespiratory fitness, physical activity, and BMI with arterial health in middle‐aged men and women

**DOI:** 10.14814/phy2.14438

**Published:** 2020-05-22

**Authors:** Eero A. Haapala, Earric Lee, Jari A. Laukkanen

**Affiliations:** ^1^ Faculty of Sport and Health Sciences University of Jyväskylä Jyväskylä Finland; ^2^ Physiology, Institute of Biomedicine School of Medicine University of Eastern Finland Kuopio Finland; ^3^ Department of Internal Medicine Central Finland Healthcare District Jyväskylä Finland; ^4^ Institute of Public Health and Clinical Nutrition University of Eastern Finland Kuopio Finland

**Keywords:** Arterial stiffness, Exercise, Fitness, Obesity

## Abstract

We investigated the associations of cardiorespiratory fitness (CRF), physical activity (PA) with regard to aerobic and resistance training, and body mass index (BMI) with pulse wave velocity (PWV) and augmentation index (AIx) in middle‐aged adults with at least one cardiovascular risk factor. A total of 84 (46 men and 38 women) participated in the study. Cardiorespiratory fitness was measured using a maximal graded exercise test on a cycle ergometer and was defined as maximal power output (W_max_) normalized for body weight^‐0.35^. Participation in aerobic and resistance training was assessed by a detailed questionnaire and BMI was calculated as weight (kg)/[height (m^2^)]. Pulse wave velocity and AIx were measured using an applanation tonometry before (pre), immediately after (post), and after 10 min (post10) of maximal exercise test. Cardiorespiratory fitness, PA, or BMI was not associated with PWV or AIx. Pulse wave velocity decreased significantly from pre to post10 among those in the highest third of CRF (mean difference=−0.793 m/s, 95% CI = −1.494 to −0.091, *p* = .023) and in normal weight participants (*p* = .084 for time*group interaction mean difference=−0.781 m/s, 95% CI = −1.496 to −0.066, *p* = .029), but not among those in the other thirds of CRF or overweight or obese participants. Participants who had regular resistance training had continuously higher AIx from pre to post10 than those who had no regular resistance training (mean difference = −1.98, 95% CI = −4.02 to 0.069, *p* = .058). In conclusion, exercise may decrease PWV and AIx. Our results suggest that positive arterial responses to exercise could be slightly improved in fit and normal weight individuals.

## Graphical abstract

Arterial stiffness and impaired arterial dilatation capacity are one of the first signs of cardiovascular diseases. We found that exercise may decrease arterial stiffness and improve arterial dilatation. Our study also suggests that cardiovascular responses to exercise may be improved in fit and normal weight individuals.

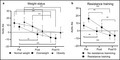

## 1 INTRODUCTION

Arterial stiffness is an independent risk factor for cardiovascular diseases (Vlachopoulos, Aznaouridis, & Stefanadis, 2010); which are still the leading cause of morbidity and premature mortality worldwide (Global status report on noncommunicable diseases:, 2014, 2015). Stiffening of arterial wall decreases compliance and distensibility of arteries in response to pressure changes and increases pulse pressure. This may lead to pathophysiological left ventricular hypertrophy, and an increased risk of hypertension, ischemic heart disease, and heart failure (Zieman, Melenovsky, & Kass, 2005). Arterial stiffness develops through complicated cellular and molecular processes leading to frayed and broken elastin, and increases what is often unorganized and dysfunctional collagen fiber content in the artery wall (Zieman et al., 2005).

While arterial stiffness increases naturally with increasing age (McEniery, Yasmin, Qasem, Wilkinson, & Cockcroft, 2005), it has been suggested that low cardiorespiratory fitness, lack of physical activity, obesity, and being overweight leads to early vascular aging and premature increases in arterial stiffness (Nilsson, 2008). Higher cardiorespiratory fitness has been inversely associated with lower arterial stiffness in children (Veijalainen et al., 2016), adolescents and young adults (Boreham et al., 2004; Haapala, Laukkanen, Takken, Kujala, & Finni, 2018), and middle‐aged and older adults (Vaitkevicius et al., 1993). Previous studies in adults have investigated the associations of measures of cardiorespiratory fitness scaled by body weight (BW); yet simple ratio scaling by BW has been found to be physiologically and statistically inappropriate method to remove the effect of body size on cardiorespiratory fitness (Krachler et al., 2015; Tanner, 1949; Welsman & Armstrong, 2019). Therefore, use of ratio standard scaling may have obscured our understanding on the role of cardiorespiratory fitness in the development of arterial stiffening.

Physical activity and particularly vigorous intensity physical activity has been inversely associated with arterial stiffness in cross‐sectional (Endes et al., 2016) and longitudinal studies ( Laar et al., 2011). Exercise interventions have also provided some evidence that regular aerobic exercise (Ashor, Lara, Siervo, Celis‐Morales, & Mathers, 2014; Li et al., 2015), but not resistance exercise (Miyachi, 2013), may decrease arterial stiffness, but the positive effect of aerobic exercise training on arterial stiffness has not been confirmed in all studies (Montero, Roche, & Martinez‐Rodriguez, 2014). Furthermore, studies on the role of cardiorespiratory fitness, body mass index (BMI), or blood pressure in the effects of acute maximal exercise on arterial stiffness and arterial dilatation capacity remain somewhat limited.

The role of cardiorespiratory fitness, exercise training, and weight status in arterial stiffness and arterial tone is still limited and inconsistent. Therefore, the aim of this study was to investigate the role of cardiorespiratory fitness normalized for BW using allometry and ratio standard, endurance and resistance exercise, BMI, and blood pressure with arterial stiffness and arterial tone. We also studied the associations of cardiorespiratory fitness, endurance and resistance exercise, and BMI with blood pressure. Finally, we investigated the role of cardiorespiratory fitness, endurance and resistance exercise, BMI, and blood pressure on changes in arterial stiffness and arterial dilatation tone in response to a maximal graded exercise test. We hypothesized that cardiorespiratory fitness, endurance exercise training, and overweight and obesity, and elevated blood pressure are associated with poorer arterial health in adults with at least one cardiovascular risk factor.

## 2 METHODS

### 2.1 Participants

A total of 102 participants were recruited from the city of Jyväskylä, Central Finland region, through the local out‐of‐hospital health care center. The study group consisted of asymptomatic participants (no cardiovascular symptoms) with at least one cardiovascular risk factor, such as a history of smoking, dyslipidemia, hypertension, obesity, diabetes, or family history of coronary heart disease. All participants with diagnosed cardiovascular diseases were excluded. Prior to participation in the study, all participants were informed about the research purposes and measurement procedures and were screened by a cardiac specialist. A total of 84 (46 men and 38 women) had the complete data used in these analyses. The research protocol and study design were approved by the institutional review board of the Central Finland Hospital District ethical committee, Jyväskylä, Finland (Dnro 5U/2016). All study participants provided written informed consent prior to the inclusion in the study. The study was performed as stated by the Declaration of Helsinki.

### 2.2 Assessment of cardiorespiratory fitness

A maximal exercise test was conducted on an electromagnetically braked cycle ergometer (Monark Exercise AB, Sweden) utilizing a graded exercise test protocol with continuous ECG recordings (CardioSoft software V.1.84, GE Healthcare, Freiburg, Germany) to assess the level of aerobic exercise capacity (Laukkanen et al., 2018). The symptom‐limited exercise test was started with 3‐min warm‐up without workload for each participant and continued with 20 W increments applied every 1 min until volitional exhaustion. All exercise tests were supervised by an experienced physician with the assistance of a trained nurse.

We defined cardiorespiratory fitness as the maximal power output (W_max_) defined as the workload at the end of the exercise test scaled by BW^‐1^ and allometrically scaled W_max_ by BW^‐0.35^, because W_max_/BW^‐1^ had a strong inverse association with BW (β=−0.587, 95% confidence interval (CI) = −0.782 to −0.392, *p* < .001) indicating that ratio scaling by BW^‐1^ was not able to remove the effect of body size on cardiorespiratory fitness. Therefore, allometric scaling of W_max_ was performed by log‐linear regression models (Agbaje et al., 2019). The scaling exponent for BM was 0.35 (95% CI = 0.14 to 0.56). The power function ratio removed the associations of W_max_ with BW (β = −0.061, 95% CI = −0.317 to 0.195, *p* = .636) suggesting the validity in scaling cardiorespiratory fitness for body size.

### 2.3 Assessment of physical activity

We assessed aerobic and resistance exercise by a questionnaire (Lakka et al., 2010). The participants filled in a questionnaire whether they participated in aerobic or resistance exercise. This is a detailed quantitative questionnaire based on common physical activities and enables the assessment of key components of physical activity. Participants were asked to record the frequency (number of sessions per week), average duration (hours and minutes per session), and intensity of their physical activity. Because of missing data on the frequency, average duration, and the intensity of physical activity, we divided participants as those who participated in aerobic or resistance training and those who did not.

### 2.4 Assessment of arterial stiffness and blood pressure

Pulse wave velocity (PWV) and augmentation index (AIx) as measures of arterial stiffness were measured before, immediately after, and 10 min after the maximal graded exercise test by a single trained researcher (EL) using a PulsePen device (DiaTecne s.r.l., Milan, Italy; http://www.pulsepen.com) which includes one tonometer and an integrated ECG unit (Lee et al., 2018, 2019). The measurement of arterial stiffness and arterial dilatation capacity followed closely established guidelines (Tomlinson, 2012; Van Bortel et al., 2012).

Pulse wave velocity was measured by recording carotid and peripheral (femoral) waveforms in rapid succession at a sample rate of 1 kHz, and defined as the transit distance between the measuring sites divided by the time delay between the distal pulse and proximal pulse wave, using the ECG trace as reference. Transit distances were assessed by body surface measurements using a tape measure from the suprasternal notch to each pulse recording site (carotid and femoral). Direct carotid to femoral measurement was adjusted to 80% (common carotid artery–common femoral artery × 0.8) for the calculation of PWV as recommended by current guidelines. Transit time was defined as the difference between the delay of the distal pulse wave to the R wave belonging to the ECG qRs complex and the delay of the proximal pulse wave to the R wave belonging to the ECG qRs complex. The pulse wave delay was determined by calculating the time elapsed from the peak of the R wave and the “foot” of the pressure pulse wave.

Augmentation index is another measure that has been used to describe central arterial stiffness but AIx also reflects peripheral arterial tone. Changes in AIx in response to exercise may reflect arterial dilatation and opening of peripheral arterial tree. AIx was obtained from the carotid pressure waveform analysis. AIx is a parameter which provides an indication of the contribution of reflected waves to the total pulse pressure and was defined as the difference between the second and first systolic peak on arterial pulse waveform and was expressed as a percentage of central pulse pressure ((AIx)=augmented pressure/pulse pressure × 100).

Supine brachial systolic blood pressure and diastolic blood pressure were obtained using an oscillometric Microlife BP A200 blood pressure monitor (Microlife Corp., Taipei, Taiwan) for better sensitivity and accuracy. Two sequential readings were recorded, and the mean values were used. Participants rested in the supine position for 10 min before PWV was measured at baseline. However, due to the nature of the study, arterial stiffness was measured immediately and 10 min after the exercise test exposure without having laid supine for 10 min.

The pressure values recorded by tonometry were calibrated to the blood pressure values obtained at the brachial artery; where they were assigned to the appropriate pixels and the values for mean arterial pressure and all other pressure‐related parameters were re‐established. The values deduced by the software apply the established concept that the mean arterial pressure remains unchanged in the tract from the aorta to the peripheral arteries. Mean arterial pressure was calculated by the software as diastolic blood pressure + 1/3(systolic blood pressure–diastolic blood pressure).

### 2.5 Statistical methods

Statistical analyses were performed by SPSS statistical software, version 23.0 (IBM corp. Armonk, NY, USA). Basic characteristics between sexes were compared using Student's *t* test for normally distributed continuous variables, the Mann–Whitney *U* test for skewed continuous variables, or chi‐squared test for categorical variables. The associations of cardiorespiratory fitness and BMI with PWV, AIx, systolic and diastolic blood pressure, and mean arterial pressure were investigated using linear regression analyses adjusted for age and sex. Linear regression analyses were also used to investigate the associations of systolic and diastolic blood pressure, and mean arterial pressure with PWV and AIx. Differences in PWV and AIx between those who regularly engaged in aerobic or resistance training and those who did not were studied with ANOVA adjusted for age and sex. The associations of cardiorespiratory fitness, BMI, aerobic exercise, and resistance exercise with PWV were further adjusted for mean arterial pressure and those with AIx for heart rate (Wilkinson et al., 2000) and mean arterial pressure.

We studied changes in PWV and AIx in response to a maximal graded exercise test and the role of cardiorespiratory fitness, BMI, exercise, and blood pressure in these changes using repeated measures ANOVA. In these analyses, we used only W_max_/BW^‐0.35^ as a measure of cardiorespiratory fitness because it efficiently removed the effect of body size on cardiorespiratory fitness. We investigated the role of cardiorespiratory fitness as thirds, weight status (normal weight, overweight, obesity), aerobic and resistance exercise (yes/no; N for resistance exercise = 82), blood pressure (normotensive (systolic blood pressure < 120 mmHg and diastolic blood pressure < 80 mmHg), high (systolic blood pressure 120–129 mmHg and diastolic blood pressure < 80 mmHg), stage 1 (systolic blood pressure 130–139 mmHg or diastolic blood pressure 80–89 mmHg), stage 2 (systolic blood pressure ≥ 140 mmHg or diastolic blood pressure ≥ 90 mmHg) (Whelton et al., 2018)), and PWV at the preassessment as thirds by studying time*group interactions from pre to post10 assessments using repeated measure ANOVA. Differences in PWV and AIx from pre to post10 assessments between adults in the thirds of cardiorespiratory fitness, between normal weight, overweight, and obese adults, between adults in the aerobic or resistance exercise groups, between adults in the blood pressure groups, and between those in the PWV thirds were investigated using repeated measures ANOVA with Sidak correction for multiple comparisons. These data were further adjusted for age and sex. *p* < .05 was considered statistically significant.

## 3 RESULTS

### 3.1 Basic characteristics

Men were taller and heavier, had higher blood pressure, lower AIx, and higher cardiorespiratory fitness expressed as W_max_/BW^‐0.35^ and W_max_/BW^‐1^ than women (Table 1). Men also participated in resistance training less frequently than women.

**Table 1:** Basic characteristics

	All (*n* = 84)	Men (*n* = 46)	Women (*n* = 38)	*p*
Age	53.1 (9.9)	53.3 (11.1)	52.9 (8.3)	.849
Height	172.7 (9.1)	179.0 (6.5)	165.1 (5.0)	<.001
Weight	81.4 (15.7)	88.0 (13.3)	73.4 (14.8)	<.001
Body mass index	27.2 (4.4)	27.5 (3.9)	26.9 (4.9)	.564
Weight status				.502
Normal weight (%)	31.0	28.3	34.2	
Prevalence of overweight (%)	46.4	52.2	39.5	
Prevalence of obesity (%)	22.6	19.6	26.3	
Systolic blood pressure	137.3 (15.6)	140.7 (14.0)	133.2 (16.7)	.027
Diastolic blood pressure	84.8 (10.3)	87.0 (10.7)	82.0 (9.3)	.026
Mean arterial pressure	102.3 (11.4)	104.9 (11.1)	99.1 (11.0)	.018
Aortic pulse wave velocity	9.7 (2.1)	9.9 (2.0)	9.3 (2.0)	.172
Aortic augmentation index	10.9 (14.2)	6.3 (13.7)	16.6 (12.8)	.001
Maximal power output (W)	229.9 (53.0)	261.6 (45.4)	191.6 (32.1)	<.001
Maximal power output (W/kg of BW^−0.35^)	49.3 (10.2)	54.7 (9.2)	42.8 (6.9)	<.001
Maximal power output (W/kg of BW^−1^)	2.9 (0.6)	3.0 (0.6)	2.7 (0.5)	.008
Maximal heart rate achieved in the exercise test	173.5 (13.9)	176.0 (15.7)	170.5 (10.7)	.961
Endurance exercise (%)	70.4	65.9	75.7	.338
Resistance training (%)	42.7	31.8	55.3	.032

Data are from the Student's *t* test for continuous variables and chi‐squared test for categorical variables and are displayed as means (*SD*) or percentages (%). *p* values refer to statistical significance for differences between men and women. BW = body weight.

### 3.2 Associations of cardiorespiratory fitness, physical activity, and BMI with arterial stiffness, arterial dilatation capacity, and blood pressure

Cardiorespiratory fitness expressed as W_max_/BW^‐0.35^ or W_max_/BW^‐1^ were not associated with PWV or AIx after adjustment for age and sex (Table 2). Further adjustment for mean arterial pressure or heart rate had no effect on these associations. Nevertheless, cardiorespiratory fitness expressed as W_max_/BW^‐1^ was positively associated with PWV in women (*β* = 0.253, *p* = .145) but inversely associated with PWV in men (*β* = −0.256, *p* = .056; *p* = .019 for interaction). Further adjustment for mean arterial pressure attenuated the association between W_max_/BW^‐1^ and PWV in men (β‐0.121, *p* = .230). In addition, cardiorespiratory fitness expressed as W_max_/BW^‐1^ was inversely associated with diastolic blood pressure after adjustment for age and sex. However, cardiorespiratory fitness was not associated with diastolic blood pressure when it appropriately expressed as W_max_/BW^‐0.35^.

**Table 2:** Associations of cardiorespiratory fitness, exercise, and body mass index with arterial stiffness and dilatation capacity and blood pressure. *N* = 84

	Aortic pulse wave velocity		Augmentation index		Systolic blood pressure		Diastolic blood pressure		Mean arterial pressure	
	β (95% CI)	*p*	β (95% CI)	*p*	β (95% CI)	*p*	β (95% CI)	*p*	β (95% CI)	*p*
Maximal power output/ BW^−0.35^	−0.047 (−0.309 to 0.216)	.725	0.033 (−0.227 to 0.293)	.799	0.049 (−0.213 to 0.311)	.710	−0.031 (−0.302 to 0.239)	.818	0.003 (−0.262 to 0.267)	.982
Maximal power output/ BW^−1^	−0.051 (−267 to 0.267)	.639	0.023 (−0.191 to 0.237)	.831	−0.082 (−0.294 to 0.133)	.450	−0.223 (−0.440 to −0.005)	.045	−0.172 (−0.387 to 0.042)	.113
Body mass index	0.002 (−0.200 to 0.204)	.986	0.007 (−0.193 to 0.208)	.942	0.123 (−0.077 to 0.324)	.224	0.325 (0.129 to 0.520)	.001	0.253 (0.057 to 0.448)	.012
Systolic blood pressure	0.494 (0.301 to 0.688)	<.001	0.357 (0.152 to 0.563)	.001						
Diastolic blood pressure	0.496 (0.311 to 0.682)	<.001	0.414 (0.222 to 0.607)	<.001						
Mean arterial pressure	0.538 (0.352 to 0.352)	<.001	0.424 (0.226 to 0.621)	<.001						

Note

The data are standardized regression coefficients and their 95% confidence intervals adjusted for age and sex. BW = body weight.

Sex modified the associations of resistance training and PWV (*p* = .036 for sex interaction), but the differences between women (9.8 vs. 8.8 m/s, mean difference = 1.0 m/s, 95% CI = −0.279 to 2.281, *p* = .121) or men (9.3 vs. 10.1 m/s, mean difference = −0.820 m/s, 95% CI = −2.002 to 0.361, *p* = .161) who resistance trained and those who did not were not statistically significant. Further adjustment for mean arterial pressure augmented the differences in men (9.2 vs. 10.1 m/s, mean difference=−0.864 m/s, 95% CI = −1.650 to −0.078, *p* = .032), but it had no effect on the differences in women (data not shown). Participants who resistance trained had higher AIx than those who did not (14.9% vs. 8.0%, mean difference 6.8%, 95% CI = 1.1 to 12.6, *p* = .020). This difference remained statistically significant after further adjustment for heart rate. There were no other statistically significant differences between aerobic or resistance training groups.

Body mass index was not associated with PWV or AIx in men and women combined. However, BMI was positively associated with PWV in men (*β* = 0.264, *p* = .048) and inversely with PWV in women (*β*=−0.247, *p* = .121, *p* = .012 for interaction). Further adjustment for mean arterial pressure strengthened the association in women (*β* = −0.317, *p* = .034) but attenuated it in men (*β* = −0.037, *p* = .730). Systolic blood pressure, diastolic blood pressure, and mean arterial pressure were directly associated with PWV and AIx (Table 2).

### 3.3 Effects of cardiorespiratory fitness, aerobic and resistance training, and weight status on changes in arterial stiffness in response to a maximal graded exercise test

Pulse wave velocity decreased from pre‐ to post‐test (mean difference = −0.420 m/s, 95% CI = −0.893 to 0.052, *p* = .096) but was higher at post10 than preassessment (mean difference (pre vs. post10) = 0.258 m/s, 95% CI = −0.494 to 0.258, *p* = .043 for main effect).

**Figure 1:**
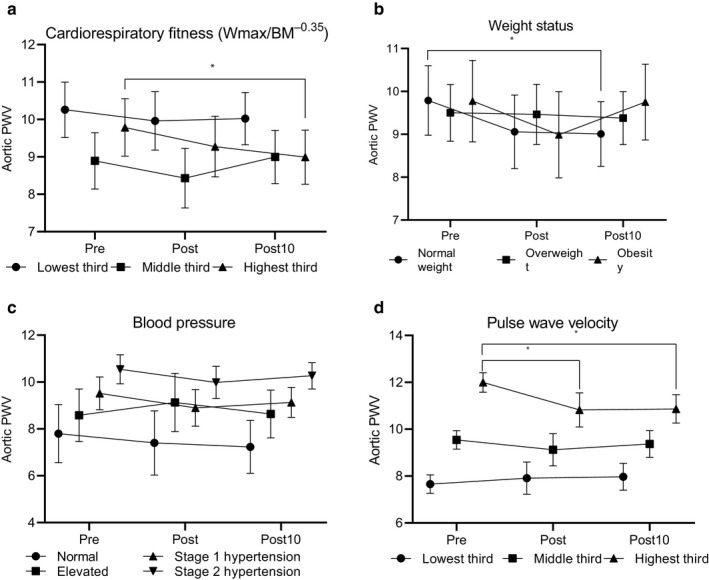
Changes in aortic pulse wave velocity (PWV) from pre‐ to post‐ and post10‐exercise assessments among adults in the thirds of cardiorespiratory fitness expressed as maximal power output (Watts) scaled by body weight in kilograms^0.35^ (N for the lowest, middle, and the highest third were 29, 28, and 27, respectively) (a), normal weight (*N* = 26), overweight (*N* = 39), and obese (*N* = 19) adults (b), adults with normal blood pressure (*N* = 9), with elevated blood pressure (*N* = 11), with stage 1 hypertension (*N* = 28), and stage 2 hypertension (*N* = 36) (c), and adults in the thirds of PWV (N for the lowest, middle, and the highest third were 29, 29, and 26, respectively) (d). Data are mean and 95% confidence interval. * denotes statistically significant (*p* < .05) change in PWV

Cardiorespiratory fitness expressed as W_max_/BW^‐0.35^ did not modify the changes in PWV in response to exercise (*p* = .196 for W_max_/BW^‐0.35^*time interaction, Figure 1a). However, participants in the lowest third of W_max_/BW^‐0.35^ had continuously higher PWV from pre to post10 than those in the second third (mean difference 1.3 m/s, 95% CI = 0.1 to 2.5, *p* = .022, *p* = .027 for the main effect). This difference was no longer statistically significant after further adjustment for age and sex (*p* = .462). Further adjustment for mean arterial pressure strengthened these differences (p for the main effect = 0.043), but the post hoc comparisons were not statistically significant. Additional analyses showed that PWV decreased significantly from pre to post10 only in the highest third of W_max_/BW^‐0.35^ (mean difference=−0.793 m/s, 95% CI=−1.494 to −0.091, *p* = .023) after adjustment for age and sex.

Weight status modified the effects of a maximal graded exercise test on PWV (*p* = .084 for interaction, Figure 1b). Pulse wave velocity decreased from pre to post10 assessment (mean difference=−0.781 m/s, 95% CI=−1.496 to −0.066, *p* = .029) only in normal weight participants (*p* = .034 for the main effect). There were no differences in PWV among normal weight, overweight, and obese participants (*p* = .914 for the main effect). Additional adjustment for mean arterial pressure had no effect on these results.

Resting blood pressure did not modify the exercise response on PWV (*p* = .930 for interaction, Figure 1c). Normotensive participants had lower PWV from pre to post10 than those at stage 1 (mean difference=−1.70, 95% CI=−3.42 to −0.11, *p* = .052) and stage 2 hypertension (mean difference=−2.79, 95% CI=−4.45 to −1.12, *p* < .001, *p* < .001 for main effect).

Pre‐PWV modified the effect of a maximal graded exercise test on PWV (*p* = .003 for interaction, Figure 1d). Pulse wave velocity decreased only in highest third from pre to post (mean difference = −1.2, 95% CI = −2.2 to −0.1, *p* = .026) and post10 (mean difference = −1.1, 95% CI = −1.9 to −0.4, *p* = .003). Participants who were in the highest third of PWV at the preassessment had continuously higher PWV from pre to post10 than those the middle (mean difference = 1.9, 95% CI = 1.1 to 2.7, *p* = .003) and the lowest third (mean difference = 3.4, 95% CI = 2.6 to 4.2, *p* < .001, *p* < .001 for main effect). Further adjustments had no effect on these results.

Aerobic endurance training or resistance training did not modify PWV responses to exercise. There were no statistically significant differences in PWV between those who exercised regularly and those who did not.

### 3.4 Effects of cardiorespiratory fitness, aerobic and resistance training, and weight status on changes in arterial tone in response to a maximal graded exercise test

AIx decreased linearly from pre to post10 (mean difference pre vs. post=−9.2%, 95% CI = −13.7 to −4.7, *p* < .001; post vs. post10 = −8.0%, 95% CI = −12.6 to 3.5, *p* < .001 for main effect).

**Figure 2:**
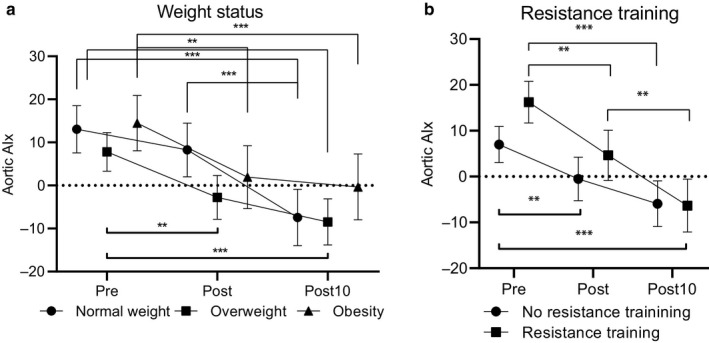
Changes in augmentation index (AIx) from pre‐ to post‐ and post10‐exercise assessments among (a), normal weight (*N* = 26), overweight (*N* = 39), and obese (*N* = 19) adults, and (b), adults with (*N* = 35) and without (*N* = 47) regular resistance training. Data are mean and 95% confidence interval. *(*p* < .05), ** (*p* < .01), and *** (*p* < .001) denotes statistically significant change in AIx

Weight status partly modified the effect of a maximal graded exercise test on AIx (*p* = .062 for interaction, Figure 2a). In normal weight participants, AIx at post10 was lower than in pre (mean difference = −20.5, 95% CI = −27.2 to −13.8, *p* < .001) and post (mean difference = −15.7, 95% CI = −22.8 to −8.6, *p* < .001) (*p* < .001 for the main effect). In overweight participants, AIx at post (mean difference = −10.6, 95% CI = −17.9 to −3.2, *p* = .003) and post10 (mean difference = −16.3, 95% CI = −23.0 to −9.6, *p* < .001, *p* < .001 for main effect) were lower than pre‐exercise AIx. Similarly, in obese participants post (mean difference = −12.5, 95% CI = −21.2 to −3.9, *p* = .004) and post10 (mean difference = −14.8, 95% CI = −23.5 to −6.2, *p* = .001, *p* < .001 for main effect) AIx were lower than pre‐exercise AIx. Further adjustment for heart rate had no effect on these results.

Resistance training modified the response of AIx to a maximal graded exercise test (*p* = .026 for time*group interaction, Figure 2b) and the decrease in AIx was larger in participants with regular resistance training. AIx decreased from pre to post (mean difference = −11.6, 95% CI = −18.8 to −4.4, *p* = .001) and post10 (mean difference = −22.6, 95% CI = −29.1 to −16.1, *p* < .001) in participants who had regular resistance training. AIx at post was also higher than at post10 (mean difference = 11.0, 95% CI = 3.4 to 18.5, *p* = .002). Among those who had no regular training AIx at pre was higher than at post (mean difference = 7.5, 95% CI = −1.8 to 13.2, *p* = .006) and post10 (mean difference = −12.9, 95% CI = 7.9 to 18.0, *p* < .001). Furthermore, participants who had regular resistance training had continuously higher AIx from pre to post 10 than those who had no regular resistance training (*p* = .058 for the main effect, mean difference = −1.98, 95% CI = −4.02 to 0.069, *p* = .058). This difference strengthened after adjustment for age and sex (*p* = .037). Further adjustment for heart rate had no effect on these differences. Aerobic endurance training did not modify AIx responses to exercise.

W_max_/BW^‐0.35^, blood pressure, or PWV did not modify the response of AIx to a maximal graded exercise test and there were no differences in AIx between the thirds of W_max_/BW^‐0.35^, blood pressure groups, or thirds of PWV. The results on the associations of blood pressure with AIx remained similar when normotensive and elevated groups were combined.

## 4 DISCUSSION

The most consistent finding in our study was that a higher systolic blood pressure, diastolic pressure, and mean arterial pressure were strongly associated with higher PWV and AIx. We found no association of cardiorespiratory fitness or the level of exercise training with arterial stiffness or arterial tone in middle‐aged subjects with cardiovascular risk factors. Nevertheless, we observed an inverse association between cardiorespiratory fitness expressed as W_max_/BW^‐1^ and diastolic blood pressure. However, this association attenuated when cardiorespiratory fitness was appropriately normalized for BM using allometry and expressed as W_max_/BW^‐0.35^. We also observed that a higher BMI was related to higher diastolic blood pressure and mean arterial pressure. Furthermore, PWV decreased among participants with higher fitness level and normal body weight in response to a maximal graded exercise with increasing workloads. Finally, we observed a slightly augmented AIx response to a maximal graded exercise test in normal weight compared to obese participants.

In contrast with the results of some previous studies showing an inverse association between cardiorespiratory fitness and arterial stiffness in children (Veijalainen et al., 2016), adolescents and young adults (Boreham et al., 2004; Haapala et al., 2018), and older adults (Vaitkevicius et al., 1993), we found no relationship of cardiorespiratory fitness to arterial stiffness and arterial tone. However, our results agree with findings showing weak association between cardiorespiratory fitness and arterial stiffness in young and middle‐aged adults while showing an inverse association in older adults (Vaitkevicius et al., 1993). One reason for weak associations between cardiorespiratory fitness and arterial stiffness may be that our study population had no diagnosed cardiovascular diseases, which may lead to arterial stiffening or endothelial dysfunction. Therefore, it is possible that the associations of cardiorespiratory fitness and exercise with arterial health are stronger in individuals with more advanced chronic diseases (Green, Spence, Halliwill, Cable, & Thijssen, 2011). Furthermore, previous studies have also suggested that relatively higher exercise load is required to improve vascular functions in asymptomatic healthy adults (Green et al., 2011). Another reason may be that our study sample was quite homogenous and there were not enough variation and therefore studies in larger samples are needed. Furthermore, as expected, we observed that cardiorespiratory fitness expressed as W_max_/BW^‐1^ was inversely associated with diastolic blood pressure. However, this association was no longer seen when the effect of body size on cardiorespiratory fitness was appropriately controlled using allometry. This observation suggests that the association between cardiorespiratory fitness and diastolic blood pressure was largely explained by differences in body size and composition.

We found that regular aerobic endurance exercise was not associated with arterial stiffness. Previous studies have shown that aerobic exercise training can improve vascular functions (Green et al., 2011; Thijssen et al., 2010). However, aerobic exercise training has been found to have only limited effects of exercise training on arterial stiffness in obese (Montero, Roberts, & Vinet, 2014) or prehypertensive or hypertensive adults (Montero, Roche, et al., 2014). Therefore, it is possible that the effects of aerobic exercise training on arterial stiffness require substantial reduction also in other cardiometabolic risk factors (Montero, Roche, et al., 2014). Another explanation for these contrasting findings may be that we used a rough dichotomy assessment method to separate those who have a history of regular aerobic exercise. Second, our methodology for regular physical activity was not detailed enough to assess intensity of exercise. It is possible that the reported regular aerobic exercise may have been of too low in intensity and/or duration to cause beneficial effects on arterial stiffness in this population with cardiovascular risk factors. Indeed, shear stress against the artery wall has been shown to be an important factor for improving vascular function (Green et al., 2011). While there is evidence showing that strength training has none or increasing effects on arterial stiffness (Miyachi, 2013), we found some evidence on the dimorphic associations according to gender between strength training groups in PWV showing that women who resistance trained had stiffer arteries than those who did not trained while opposite was observed in men, although these differences did not reach statistical significance. The reason for these opposing associations is not well understood. Menopause has been found to amplify arterial stiffening (Takahashi et al., 2005), and it is possible that women who preferred resistance training were already menopausal and they were encouraged to regularly participate in a resistance training program. However, possible sex‐differences in these associations warrants further studies.

We observed that BMI was related to diastolic blood pressure and mean arterial pressure, but not with arterial stiffness. Furthermore, in line with previous studies (Safar et al., 2018) blood pressure was strongly and directly associated with PWV and AIx. Increased blood pressure has been found to decrease elastic potential of artery wall by causing excessive collagen production (Zieman et al., 2005), and change vascular smooth muscle cell action, thereby (Safar et al., 2018) increasing arterial stiffness. In addition, increased arterial stiffness may partly explain elevated blood pressure (Safar et al., 2018).

A graded exercise with increasing workload had a small overall effect on PWV. However, we found that those participants with the highest cardiorespiratory fitness, normal weight, and those with the highest PWV demonstrated a slight decrease in PWV in response to a maximal graded exercise test. We also found a slight decrease in PWV in obese participants from pre to post, but PWV returned to pre levels within 10 min. These results may imply that individuals with higher cardiorespiratory fitness and normal weight can better regulate vascular function and blood conductance to match peripheral oxygen requirements. Previous studies have shown that sedentary individuals have blunted nitric oxide‐dependent vasodilatation that can be prevented by exercise (Black, Green, & Cable, 2008).

Furthermore, obesity has been associated with impaired nitric oxide‐dependent vasodilatation (Higashi, Sasaki, Nakagawa, & Matsuura, 2001) suggesting that obese adults could not increase arterial dilatation in a similar manner to normal weight adults, which is an essential vascular adaption to increasing exercise workload. These observations are partly supported by our findings showing that only normal weight participants exhibited a consistent decrease AIx in response to a maximal graded exercise test. We also found that although those who resistance trained had higher AIx preassessment, the AIx decreased to the same level after a maximal graded exercise test than in other participants. A plausible explanation for decreased AIx in response to a maximal graded exercise test is exercise‐induced nitric oxide‐dependent peripheral vasodilation (Green et al., 2011). The reason for higher AIx in resistance training participants may be that resistance training induced peaks in blood pressure may have increase vascular smooth muscle tone (Safar et al., 2018).

The strengths of this study include a measurement of cardiorespiratory fitness using a maximal exercise test and valid and reproducible measures of arterial stiffness. We not only investigated cross‐sectional associations of cardiorespiratory fitness, BMI, exercise training, and blood pressure with arterial stiffness but also their role in the effects of a maximal graded exercise test, which is commonly used in clinical practice, allowing us to capture more comprehensive picture of determinants of arterial health. We also utilized allometric scaling to account the effect of body size on cardiorespiratory fitness. However, because we did not measure body composition in the present study, we were unable to normalize cardiorespiratory fitness for lean body mass which is considered the gold standard measure to remove the effect of body size and composition on cardiorespiratory fitness (Loftin, Sothern, Abe, & Bonis, 2016). Furthermore, we assessed aerobic and resistance exercise training by a questionnaire giving a rough estimate of the prevalence of exercise training in the study population. The sample size was relatively small decreasing statistical power to detect statistically significant associations and decreases generalizability of the observed results and make the interpretation of possible sex‐difference difficult. Our cross‐sectional study design using an acute exercise experiment setting does not allow us to draw a conclusion on the causality of the long‐term relationship of cardiorespiratory fitness, exercise training, BMI, or blood pressure with arterial stiffness.

In conclusion, our findings showed that elevated blood pressure was strongly related to increase arterial stiffness and tone. These results indicate that lowering blood pressure would have the largest effect on improving arterial compliance. Furthermore, we found that cardiorespiratory fitness, endurance or resistance training, or BMI were not associated with arterial stiffness. Acute graded exercise may decrease PWV and AIx and thereby improve cardiovascular health. Furthermore, a maximal graded exercise decreased arterial stiffness in individuals with higher cardiorespiratory fitness, normal weight, or higher pre‐exercise PWV but not in those with lower cardiorespiratory fitness or those who were overweight or obese. Therefore, the results of our study suggest that positive arterial responses to exercise could be slightly improved in higher fit and normal weight individuals. The role of these slightly augmented responses in future cardiovascular morbidity and mortality among normal weight individuals and those with higher fitness levels warrants further study.

## CONFLICT OF INTEREST

The authors declare no conflict of interest and have no financial relationship to disclose.

## AUTHOR CONTRIBUTION

Conceptualization; EAH, EL, and JAL; methodology and data collection: EL and JAL; writing—original draft preparation: EAH; writing—review and editing: EL and JAL. All authors have read and agreed to the submitted version of the manuscript.

## DATA STATEMENT

The datasets generated during and analyzed during the current study are available from the corresponding author on reasonable request.
